# Delayed Tetanus

**Published:** 1916-06

**Authors:** 


**Delayed Tetanus.** Brochet, Acad. de Med., Nov. 23, 1915, reports a case of a soldier who had been wounded by a shell in the right scapulo-vertebral space superficially, there being also a sinus of the right calf and of the right side of the abdomen, 4 fingers' breadths above the iliac crest. The case was attended to promptly and there was no possibility of enclosure of tetanus bacilli in the solutions used for the wounds. In addition, he had received 10 C.C. of antitetanic serum 2 hours after being wounded. Nevertheless, tetanus developed on the 97th day. R. Leriche, also reports two similar fatal cases.

